# Cathepsin C promotes microglia M1 polarization and aggravates neuroinflammation via activation of Ca^2+^-dependent PKC/p38MAPK/NF-κB pathway

**DOI:** 10.1186/s12974-019-1398-3

**Published:** 2019-01-16

**Authors:** Qing Liu, Yanli Zhang, Shuang Liu, Yanna Liu, Xiaohan Yang, Gang Liu, Takahiro Shimizu, Kazuhiro Ikenaka, Kai Fan, Jianmei Ma

**Affiliations:** 1 Department of Anatomy, Dalian Medical University, West Section No.9, South Road, Lvshun, Dalian, 116044 Liaoning China; 2 Liaoning Provincial Key Laboratory of Brain Diseases, Dalian Medical University, Dalian, 116044 Liaoning China; 3 Basic Medicine College, Dalian Medical University, Dalian, 116044 Liaoning China; 4 Wolfson Institute for Biomedical Research, University College London, London, UK; 5 Division of Neurobiology and Bioinformatics, National Institute for Physiological Sciences, Okazaki, Aichi 444-8787 Japan; 6 The National and Local Joint Engineering Research Center for Drug Development of Neurodegenerative Disease, Dalian Medical University, Dalian, 116044 Liaoning China

**Keywords:** Neuroinflammation, Microglia, Cathepsin C, Cytokine, NR2B

## Abstract

**Background:**

Microglia-derived lysosomal cathepsins are important inflammatory mediators to trigger signaling pathways in inflammation-related cascades. Our previous study showed that the expression of cathepsin C (CatC) in the brain is induced predominantly in activated microglia in neuroinflammation. Moreover, CatC can induce chemokine production in brain inflammatory processes. In vitro studies further confirmed that CatC is secreted extracellularly from LPS-treated microglia. However, the mechanisms of CatC affecting neuroinflammatory responses are not known yet.

**Methods:**

CatC over-expression (CatCOE) and knock-down (CatCKD) mice were treated with intraperitoneal and intracerebroventricular LPS injection. Morris water maze (MWM) test was used to assess the ability of learning and memory. Cytokine expression in vivo was detected by in situ hybridization, quantitative PCR, and ELISA. In vitro, microglia M1 polarization was determined by quantitative PCR. Intracellular Ca^2+^ concentration was determined by flow cytometry, and the expression of NR2B, PKC, p38, IkBα, and p65 was determined by western blotting.

**Results:**

The LPS-treated CatCOE mice exhibited significantly increased escape latency compared with similarly treated wild-type or CatCKD mice. The highest levels of TNF-α, IL-1β, and other M1 markers (IL-6, CD86, CD16, and CD32) were found in the brain or serum of LPS-treated CatCOE mice, and the lowest levels were detected in CatCKD mice. Similar results were found in LPS-treated microglia derived from CatC differentially expressing mice or in CatC-treated microglia from wild-type mice. Furthermore, the expression of NR2B mRNA, phosphorylation of NR2B, Ca^2+^ concentration, phosphorylation of PKC, p38, IκBα, and p65 were all increased in CatC-treated microglia, while addition of E-64 and MK-801 reversed the phosphorylation of above molecules.

**Conclusion:**

The data suggest that CatC promotes microglia M1 polarization and aggravates neuroinflammation via activation of Ca^2+^-dependent PKC/p38MAPK/NF-κB pathway. CatC may be one of key molecular targets for alleviating and controlling neuroinflammation in neurological diseases.

## Introduction

Cysteine cathepsins have been viewed as enzymes involved in final protein degradation in the lysosomes. Recently, evidence from numerous studies has demonstrated that some cathepsins, such as cathepsin B, L, S, H, and X, play important roles in health or disease condition in the central nervous system (CNS) [1–6].

Cathepsin C (CatC), also known as dipeptidyl peptidase I, is a cysteine exopeptidase that is expressed in many types of cells. CatC functions as a key enzyme in the activation of granule serine proteases in cytotoxic T lymphocytes, natural killer cells (granzymes A and B), mast cells (chymase and tryptase), and neutrophils (cathepsin G, proteinase 3, and elastase) through removing N-terminal pro-dipeptides from the zymogen forms of these proteases [7–9]. The roles of CatC in modulating the inflammatory responses have been assessed in a number of animal models of inflammation. It has been found that CatC knockout mice are completely resistant to the acute arthritis and showed a high degree of protection in a collagen-induced rheumatoid arthritis model [10, 11]. In addition, the inflammatory cell infiltration and proinflammatory cytokine production have been shown to be decreased in CatC knockout mouse models of asthma, chronic obstructive pulmonary disease (COPD), sepsis, and abdominal aortic aneurysms [12–15]. Thus, CatC has been considered as a potential pharmacological target in inflammation therapy [16].

More recently, CatC expression is found in the normal brain in a region-dependent manner, and granular immunoreactive signals mainly exist in neuronal perikarya of particular brain regions, including the accessory olfactory bulb, the septum, CA2 of the hippocampus, a part of the cerebral cortex, the medial geniculate, and the inferior colliculus [17, 18]. In different pathological conditions, the expression of CatC is upregulated dramatically and distributed widely in the brain, for instance, strong expression of CatC is found in peri-damaged portions of hypoxic-ischemic injury [18], in the whole brain of intraperitoneally injected LPS-induced neuroinflammation [17], and in demyelinating areas in the cuprizone-induced demyelination model [19]. The cellular localization analysis in our previous study shows that CatC is induced predominantly in activated microglia (MG) in neuroinflammation. In vitro study further reveals the inducers of microglial CatC expression and secretion could be LPS or proinflammatory factors such as interleukin-1β (IL-1β) and IL-6 stimulation [17]. On the other hand, our previous study also found that CatC can induce glia-derived chemokine (CXCL2) production, which may attract inflammatory cells to sites of myelin sheath damage in a cuprizone model [19]. These lines of evidence suggest that CatC is involved in regulation of normal neuronal functions in certain brain regions, and more importantly, participates in inflammatory processes accompanying pathogenesis in the CNS. Herias et al. [20] reported that CatC may exert immunomodulatory effects on macrophages and pointed that there is an autocrine feedback of CatC in macrophage polarization towards M1 in atherosclerotic lesion. Our previous study has found that CatC is secreted extracellularly from MG following LPS stimulation [17]. Furthermore, the enzymatic activity of extracellular CatC is upregulated by the treatment of LPS, IL-6, and IL-1β. These findings implicate that secreted CatC may play functionally significant roles extracellularly.

Neuroinflammation is now recognized as a key feature in the pathogenesis of neurodegenerative diseases and neuropsychiatric disorders [21, 22]. Microglia as the principle immune cells in the CNS are activated and release inflammatory mediators capable of provoking neuroinflammation. The overactivation of microglia has been implicated in loss of memory, cognitive deficit, and behavioral impairments of many neurological diseases [22–26]. However, whether and how CatC impacts on microglial activation and inflammatory responses, further causes diminished cognition and memory, namely, the functional roles of CatC and possible molecular mechanisms in inflammation-associated brain diseases is not known yet.

In the present study, the effects of CatC on neuroinflammation was first investigated in intraperitoneal (i.p.) and intracerebroventricular (i.c.v.) LPS injection model by using conditional CatC over-expression (CatCOE) and knock-down (CatCKD) transgenic mice, and possible mechanisms involved were further studied by in vitro experiments. We found that CatC aggravates neuroinflammation by promoting MG polarization towards M1 phenotype, and this effect is mainly achieved through activation of Ca^2+^-dependent PKC/p38 MAPK/NF-κB pathway which is triggered by glutamate receptor subunit NR2B. Our study may have implications for the prevention and the treatment of inflammation-related neurological disorders.

## Material and method

### Animals

Adult C57BL/6 mice were obtained from Dalian Medical University Animal Center. CatC STOP-tetO mice (CatC STOP-tetO/+; C57BL/6 background), and Iba1-tTA mouse line 75 (initial BDF1 background backcrossed to C57BL/6 background) were kindly provided by Professor Ikenaka [27]. CatC down-expression (CatC^STOP-tetO^/^STOP-tetO^; CatCKD) was achieved by generation of STOP-tetO knockin mice in which CatC expression was blocked in hippocampal CA2 neurons and other cells in vivo. CatC over-expression (CatC^STOP-tetO/ STOP-tetO^::Iba1-tTA; CatCOE) was achieved by crossing STOP-tetO knockin mice with the microglia-selective tTA-expressing line Iba1-tTA; thus, CatC expression was restricted in Iba-1 positive cells, not in neurons.

Adult animals (8–9 weeks) weighing 20–25 g were housed in groups of five per cage with regular light/dark cycles (lights on at 8:00 a.m., lights off at 8:00 p.m.) under controlled temperature (22 ± 2 °C) and humidity (50 ± 10%), and were given standard diet and water ad libitum. All experiments were carried out in strict accordance with the requirements of Dalian Medical University guidelines for the proper care and use of laboratory animals and were approved by the Laboratory Animal Care and Use Committee of Dalian Medical University.

### Intracerebroventricular (i.c.v.) injection

LPS (*Escherichia coli*, serotype 055:B5, Sigma-Aldrich Chemical Corp., St. Louis, MO, USA) and active CatC (R&D, MN, USA) were used in the experiment. Mice were anesthesized with intraperitoneal (i.p.) injection of tribromoethanol (0.2 ml/10 g body weight), and the skull surface was cleaned with polyvidone iodine solution. Then mice were fixed to a stereotaxic apparatus (Stoelting Company, Wood Dale, IL, USA) and received right-unilateral injection of LPS (1 mg/kg) or CatC (100 ng/kg) in a volume of 2 μl. Control mice were treated with 0.9% saline vehicle in the same volume. The following coordinates were used for central injection: 0.25 mm posterior, 1.0 mm right lateral, and 2.5 mm dorsoventral to bregma.

### Morris water maze (MWM) test

Eight-week-old mice were randomly divided into control, wild type (WT), CatCOE, and CatCKD groups. Mice were administrated of LPS (100 μg/kg, i.p.). MWM test was performed for assessment of spatial learning and memory ability as described previously [28]. The equipment included a white pool (150 cm diameter, 35 cm deep) that was filled with opaque water at approximately 22 ± 1 °C which was divided into four quadrants of equal area. An escape platform (8 cm in diameter) was placed 1 cm below the surface of the water. Geometric objects with contrasting colors and shapes were set on the wall of the water tank. Mice were trained 3 times a day at 20-min intervals for 5 consecutive days. In each trial, mice were given 90 s to find the platform. If the mice did not locate the platform within the maximum time, it was guided to the platform and allowed to remain there for 10 s. Swimming was recorded by video (AnyMaze; Stoelting Co., Wood Dale, IL, USA) above the pool. Swimming speed, the escape latency, and the number of crossings over the platform location were analyzed and plotted.

### Tissue preparation

Mice were anesthesized by injection of tribromoethanol (0.2 ml/10 g, i.p.) and transcardially perfused with 4% paraformaldehyde. Brains were stored in 20% sucrose solution. Serial 18 μm sagittal sections were prepared by the cryostat microtome (Leica CM 3050 S, Leica Microsystems AG, Wetzlar, Germany) and the samples were stored at − 80 °C until biochemical estimations.

### Immunohistochemical staining

Immunohistochemical (IHC) staining was performed as described by Ma et al. [29]. Antigen retrieval was performed, and sections were blocked with 1% bovine serum albumin (BSA) for 20 min, then incubated with primary rabbit anti-Iba1 polyclonal antibody (1:500, Wako, Osaka, Japan, catalog number 019-19741) at 4 °C overnight, secondary biotinylated IgG antibody (1:200, Vector Laboratories Inc., Burlingame, CA, USA) for 2 h at room temperature. Images were taken with the Nikon digital camera system (DS-Fi1) in combination with microscopy (Nikon eclipse 80i).

### In situ hybridization

In situ hybridization (ISH) was carried out as our previous description [17]. Briefly, 18-μm-thick frozen brain sections were fixed in 4% PFA for 15 min at room temperature. After equilibration in hybridization buffer (50% formamide, 5 × SSC, 40 mg/ml salmon sperm DNA), sections were hybridized with the Digoxigenin (DIG)-labeled TNF-α (NM_013693.2, 575-1607 bp) cRNA probes in hybridization buffer overnight at 60 °C. After washing and antigen blocking, the sections were incubated with alkaline phosphatase-conjugated anti-DIG antibody (Roche, Basel, Switzerland) at room temperature 2 h. For color development, 4-nitro blue tetrazolium chloride (NBT) (Roche Diagnostic Gmbh, Mannheim, Germany) and 5-bromo-4-chloro-3-indolyl-phosphate (BCIP) (Roche Diagnostic Gmbh, Mannheim, Germany) were incubated for 8 h at room temperature. Images of the stained sections were taken by Nikon digital camera system (DS-Fi1, Nikon Corp., Tokyo, Japan) in combination with microscopy (Nikon Eclipse 80i).

### Microglia culture

Primary microglia culture was performed as described by Fan et al. [17]. Postnatal 1–2 day mouse brains were dissected and stripped of meninges, then were aseptically dissociated into single cell suspension and seeded in 10 cm culture dish with 10% fetal bovine serum (FBS) (ICN Biomedicals, Aurora, OH, USA) in Dulbecco’s modified Eagle’s medium (DMEM) (Sigma). At the 3rd and 10th day, the culture medium was changed. After 13–14 days, detached microglia were collected and plated on 6-well plates at an appropriate density and cultured at 37 °C (5% CO_2_, 95% humidity). Murine BV2 cell line was cultured in DMEM supplemented with 10% FBS, penicillin (100 U/ml), and streptomycin (100 mg/ml), and maintained at 37 °C in a humidified incubator with 5% CO_2_. Cells were sub-cultured when reaching 90% confluence.

### Quantitative real-time PCR (qRT-PCR)

Total RNA was extracted from brain tissue and primary microglia using TRlzol reagent (Life Technologies, USA) in accordance with manufacturer’s instructions. RNA concentration and purity were assessed using OD260 and OD260/OD280 ratio (Nanodrop 2000, Thermo Scientific, USA), respectively. PrimeScriptTM RT reagent kit (Takara, DRR037A) was used for cDNA production. qRT-PCR was performed using SYBR Premix Ex Taq II (TaKaRa, RR820A) containing 2 μl cDNA, 10 μl SYBR green PCR Master Mix, 0.2 μM forward primer, 0.2 μM reverse primer, and 6 μl RNAse free water in a final volume of 20 μl and analyzed with Stratagene Mx3000p (AgilentTechnologies, Santa Clara, CA, USA).The thermal cycling parameters of q-PCR includes 95 °C 10 min,1 cycle; 95 °C 15 s, 60 °C 30 s,71 °C 45 s, 40 cycles. β-actin was used as the internal standard reference, and normalized expressions of targeted genes were calculated using the comparative CT method and fold changes were calculated using the 2^−ΔΔCt^ method [30]. All primers used in this study are designed by Takara Bio Inc., China and listed in Table 1.

**Table 1:** Primer sequences used for RT-PCR analysis

Gene	Primer	
	Forward	Reverse
TNF-α	ATCCGCGACGTGGAACTG	ACCGCCTGGAGTTCTGGAA
IL-1β	GAGCACCTTCTTTTCCTTCATCTT	TCACACACCAGCAGGTTATCATC
IL-6	CAACGATGATGCACTTGCAGA	CTCCAGGTAGCTATGGTACTCCAGA
iNOS	TAGGCAGAGATTGGAGGCCTTG	GGGTTGTTGCTGAACTTCCAGTC
CD16	GCCAATGGCTACTTCCACCAC	GTCCAGTTTCACCACAGCCTTC
CD32	CCAGAAAGGCCAGGATCTAGTG	GGGAACCAATCTCGTAGTGTCTGT
NR2B	AGAACTTGGACGCTGTATTGGAGA	TGCAACAGCCAAAGCTGGA
Ptk2	AGAACTTGGACGCTGTATTGGAGA	TGCAACAGCCAAAGCTGGA
Ptger3	TGTGTGCTGTCCGTCTGTTG	CTTCTCCTTTCCCATCTGTGTCTT
Slamf8	GTGCCAATTACACTGTTCCTGATCC	GTCCAAGCACCAGTTTATGTTGTCC
Pex5l	CCAACACTTTCATATCCGTTGCTC	CTGCCTCTGGCATGGTTTCA
HPSE	ACAAACGGAGTTGTTGAAGTGAGG	AGCACTACAGACATCGGGACAGAG
β-actin	TCATCACTATTGGCAACGACG	AACAGTCCGCCTAGAAGCAC

### Enzyme-linked immunosorbent assay (ELISA)

Blood samples were collected after MWM test and centrifuged at 2000×*g* for 20 min to obtain serum samples. Microglia culture medium was collected 24 h after LPS stimulation. Proteins from brains and primary cultured microglia were extracted using RIPA lysis buffer (Keygen Biotech. Co., LTD, Nanjing, China), and concentrations of protein were determined by the BCA Protein Assay Kit (Keygen Biotech. Co., LTD, Nanjing, China). All samples were stored at − 80 °C before assay. Detection of TNF-α, IL-1β, IL-6, and IFN-γ was performed by corresponding ELISA Development Kits (Peprotech, Rehovot, Israel) in accordance with the manufacturer’s instructions. Monitor color development with an ELISA plate reader (iMark, Bio-rad, Japan) at 405 nm with wavelength correction set at 650 nm.

### Flow cytometry analysis

Primary microglia were seeded at a density of 1 × 10^6^ cells/ml and starved with DMEM with 2% FBS overnight. After being treated with 100 ng/ml CatC for 18 h, the single-cell suspension was harvested followed by centrifugation at 300×*g* for 5 min at 4 °C. After washing and blocking with Fc Block CD16/CD32 (1:100, R&D, MN, USA) for 30 min on ice, cells were incubated for 45 min in the dark room with the followed antibodies: APC-conjugated anti-Rat CD206 (10 μl/10^6^ cells; R&D, MN, USA), FITC-conjugated anti-mouse CD86 (0.125 μg/test; eBioscience, Waltham, MA, USA). Finally, the cells were resuspended in 500 μl PBS and subjected to FACSCaliber flow cytometry (BD, Franklin Lakes, USA). The data were analyzed by the Cell-Quest data analysis software (10,000 events per sample; BD, Franklin Lakes, USA). Negative isotypes were used for gating. The percentages of positive and negative cells were measured.

### Immunofluorescent staining

Primary cultured microglia were seeded on glass cover slips and treated with 100 ng/ml CatC (R&D, MN, USA) for 18 h. After being washed with PBS, cells were fixed with 4% PFA for 20 min, then incubated with blocking buffer (5% BSA and 0.1% Triton X-100; Gentihold, Beijing, China) for 30 min, primary antibodies overnight at 4 °C. Subsequently incubated with appropriate fluorochrome-conjugated secondary antibodies and 4′,6-diamidino-2-phenylindole (DAPI, 1:1000; Sigma, St. Louis, MO, USA) at room temperature for 1 h. Images were captured by laser confocal microscopy (Leica, Wetzlar, German). The following primary antibodies were used: rabbit anti-CD86 (1:100, Abcam, Cambridge, UK), goat anti-CatC (1:100; Abcam, Cambridge, UK). The secondary antibodies were labeled with 488 Alexafluor (1:100; Abcam, Cambridge, UK), and 594 Alexafluore (1:100; Proteintech, Wuhan, China).

### Western blot analysis

After primary cultured microglia and BV2 cells were treated with active CatC (100 ng/ml) alone for 12 h, or co-stimulated with E-64 (10μΜ), an inhibitor of cysteine peptidases and pre-treated MK-801 (50 μM), an antagonist of the *N*-Methyl-D-aspartate (NMDA) receptor for 1 h, the total protein was extracted using RIPA lysis buffer (KeyGEN, Nanjing, China) containing protease inhibitors (PMSF, 1:100; Biosharp, Hefei, China) and phosphatase inhibitor cocktail (1:100; MedChem Express, Shanghai, China). The protein concentration was determined by BCA Protein Assay Kit (KeyGEN, Nanjing, China), and 30 μg protein was denatured in 4×sample buffer at 95 °C for 5 min, and then loaded per lane. Then equal amount of protein samples was resolved on SDS-PAGE and transferred to nitrocellulose membrane (NC; Pall Corporation, Mexico). The NC membranes were blocked with 5% BSA before incubation overnight with the appropriate primary antibody at 4 °C. After having been washed three times for 10 min with tris-buffered saline-Tween-20 buffer (TBST), the NC membranes were incubated with fluorescence labeling secondary antibody (1:15000; LI-COR Biosciences, Lincoln, NE, USA) in 60 min at room temperature. Images were captured and quantified by Odyssey CLx Imager and Image Studio software (LI-COR Biosciences, Lincoln, NE, USA).The following primary antibodies were used: rabbit anti-NMDA (1:500; Abcam, Cambridge, UK); rabbit anti-phospho-NMDA (1:500; Cell Signaling Technology, Danvers, MA, USA); rabbit anti-phospho-PKC (1:500; Cell Signaling Technology, Danvers, MA, USA); rabbit anti-PKC (1:500; Cell Signaling Technology, Danvers, MA, USA); mouse anti-p38 (1:500; Proteintech, Wuhan, China), rabbit anti-phospho-p38 (1:500; Cell Signaling Technology, USA), rabbit anti-phospho-IκBα (1:500; Cell Signaling Technology, Danvers, MA, USA), mouse anti-IκBα (1:500; Cell Signaling Technology, Danvers, MA, USA); rabbit anti- phospho-NF-κB p65 (1:500; Cell Signaling Technology, Danvers, MA, USA); mouse anti-NF-κB p65 (1:500; Cell Signaling Technology, Danvers, MA, USA); mouse anti-GAPDH (1:5000; Proteintech, Wuhan, China).

### Intracellular Ca^2+^ measurement

Fura-3-acetoxymethyl ester (Fluo-3 AM, Solarbio, Beijing, China) was used to detect intracellular Ca^2+^ concentrations of microglia. Flow cytometry was performed in accordance with the manufacturer’s instructions. After being treated with CatC (100 ng/ml), E-64 (10μΜ), or MK-801 (50 μM, pre-treatment for 1 h) for 6 h, microglia were resuspended in Hanks balanced salt solution (HBSS) with Ca^2+^ and MgCl_2_ at 1 × 10^7^ cells/ml. The cells were incubated with 10 μM Fluo-3 AM in (HBSS, KeyGEN, Nanjing, China) for 30 min at 37 °C. Followed by centrifugation at 300×*g* for 5 min, microglia were resuspended in 500 μl Ca^2+^ free-HBSS. The accumulation of intracellular Ca^2+^ in individual cells was measured by AccuriC6 (BD, Franklin Lakes, USA) at 506 nm of excitation wavelength. The data were analyzed using the AccuriC6 analysis software.

### Microarray hybridization and data analysis

The RNA sample of primary cultured microglia with or without CatC stimulation was extracted by Trizol reagent (Life Technologies, USA). Then RNA quantity and quality were measured by NanoDrop ND-1000, and RNA integrity was assessed by standard denaturing agarose gel electrophoresis. The Whole Mouse Genome Oligo Microarray was generated for test and control samples according to the Agilent One-Color Microarray-Based Gene Expression Analysis protocol (Agilent Technology). These profiles were generated using customized 4 × 44 K oligonucleotide microarrays produced by Agilent Technologies (Palo Alto, CA, USA). The mouse whole genome microarrays covered more than 41,000 genes and transcripts. The RNA samples were amplified and microarray hybridization was performed in Agilent’s SureHyb Hybridization Chambers using the Agilent Quick Amp labeling kit. After hybridization and washing, the hybridized slides were scanned using an Agilent DNA microarray scanner. The microarray datasets were acquired in Agilent Feature Extraction Software (v11.0.0.1) and normalized in Agilent GeneSpring GX v12.1 Software which genes marked as present were chosen for further analysis. The procedure above was carried out by KangCheng Bio-Tech, Shanghai, China.

### Statistical analysis

All experimental data were analyzed using the SPSS Statistics software 19.0 (SPSS Inc., Chicago, IL, USA, 2006). Repeated measures analysis of variance (ANOVA) was used to compare the escape latency among groups. Other data were compared using one-way ANOVA. Data are presented as means ± SEM. A value of *P* ≤ 0.05 was considered significant.

## Results

### CatC aggravated LPS-induced impairments of spatial learning and memory

**Fig. 1:**
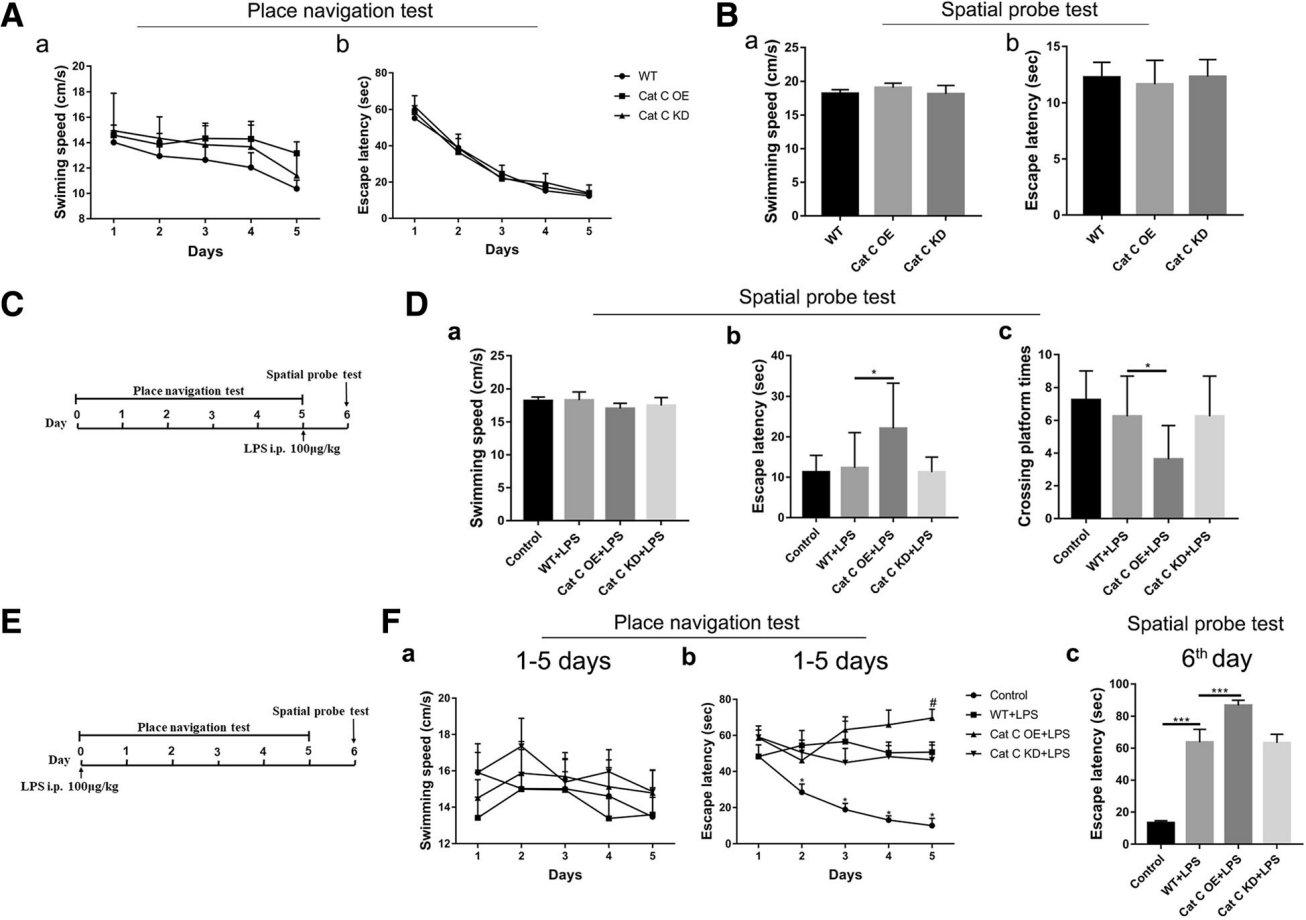
The effects of differential expression of CatC on learning and memory ability. MWM test was performed in the untreated WT, CatCOE, and CatCKD mice. Place navigation test lasted for 5 days, and spatial probe test was performed on the sixth day. **A** Learning ability of untreated mice was determined by the swimming speed (a) and escape latency (b) of place navigation test. **B** Memory ability of untreated mice was determined by swimming speed (a) and escape latency (b) of spatial probe test. **C** LPS was injected after 5-day place navigation test as shown in time axis, and spatial probe test was performed after 24 h later. **D** Memory ability of LPS-injected mice was determined by swimming speed (a), escape latency (b), and crossing platform times (c) of spatial probe test. **E** Detection of learning ability of LPS-injected mice was illustrated in time axis. **F** Learning ability of 5-day place navigation test was determined by the swimming speed (a), escape latency (b); the escape latency of the sixth day is shown in **c**. The data represent mean ± SEM, *n* = 7–9. *P ≤ 0.05, ***P ≤ 0.001. *P ≤ 0.05, 2~5th days vs. 1st day in control, #*P* ≤ 0.05, WT + LPS vs. CatCOE + LPS in Fig. 1F (b)

LPS is derived from the cell wall of Gram-negative bacteria and is a potent endotoxin that causes the release of cytokines such as interleukin-1 beta (IL-1β) and tumor necrosis factor (TNF-α). Numerous reports have demonstrated that neuroinflammation induced by a single injection of LPS or IL-1β significantly impairs hippocampal-dependent spatial learning memory by inhibiting long-term potentiation (LTP), and this impairment can be detected by Morris water maze (MWM) analysis [31–33]. In our previous study, we have demonstrated that CatC is only expressed in neurons in some specific regions of normal brain, and CatC expression in MG could be induced by a single intraperitoneal (i.p.) injection of LPS [17]. Therefore, in the present study, we still used this model to clarify the functional roles of CatC in the neuroinflammatory processes by MWM analysis. First, we performed MWM analysis in untreated CatCOE and CatCKD mice. The results showed no significant differences between the untreated WT, CatCOE, and CatCKD mice in place navigation test (Fig. 1A (a, b)) or spatial probe test (Fig. 1B (a, b)), suggesting that neither CatCOE nor CatCKD mice had changes of learning and memory function. Next, we treated another group of mice with a single injection of LPS (100 μg/kg, i.p.) after 5 days navigation test, and WT mice were treated with equivalent volume 0.9% saline as the control, then the spatial probe test was performed after 24 h of LPS administration (Fig. 1C). There were no differences in swimming speed (Fig. 1D (a)), and only CatCOE mice showed a significantly increased escape latency (Fig. 1D (b)) and reduced crossing platform times (Fig. 1D (c)), suggesting that CatCOE mice presented a serious spatial memory impairment following LPS treatment. Last, we pre-treated the third group of mice with LPS injection (100 μg/kg, i.p.) before 24 h of the place navigation test, then 5-day training and test were carried to detect learning ability (Fig. 1E). All of LPS-treated mice exhibited increased escape latency from the second day compared with the control group (Fig. 1F (a, b)). On the sixth day when learning training was finished, the escape latency in all the LPS-treated mice was significantly increased compared to control mice, and among LPS- treated mice, CatCOE mice exhibited significantly increased escape latency compared with that in wild-type or CatCKD mice (Fig. 1F (c)), suggesting CatCOE aggravated LPS-induced impairments of learning function. Taken together, the data indicate that CatC over-expression aggravated LPS-induced impairments of spatial learning and memory probably by exacerbating neuroinflammation.

### CatC aggravated peripheral and central inflammatory responses following a systemic injection of LPS

**Fig. 2:**
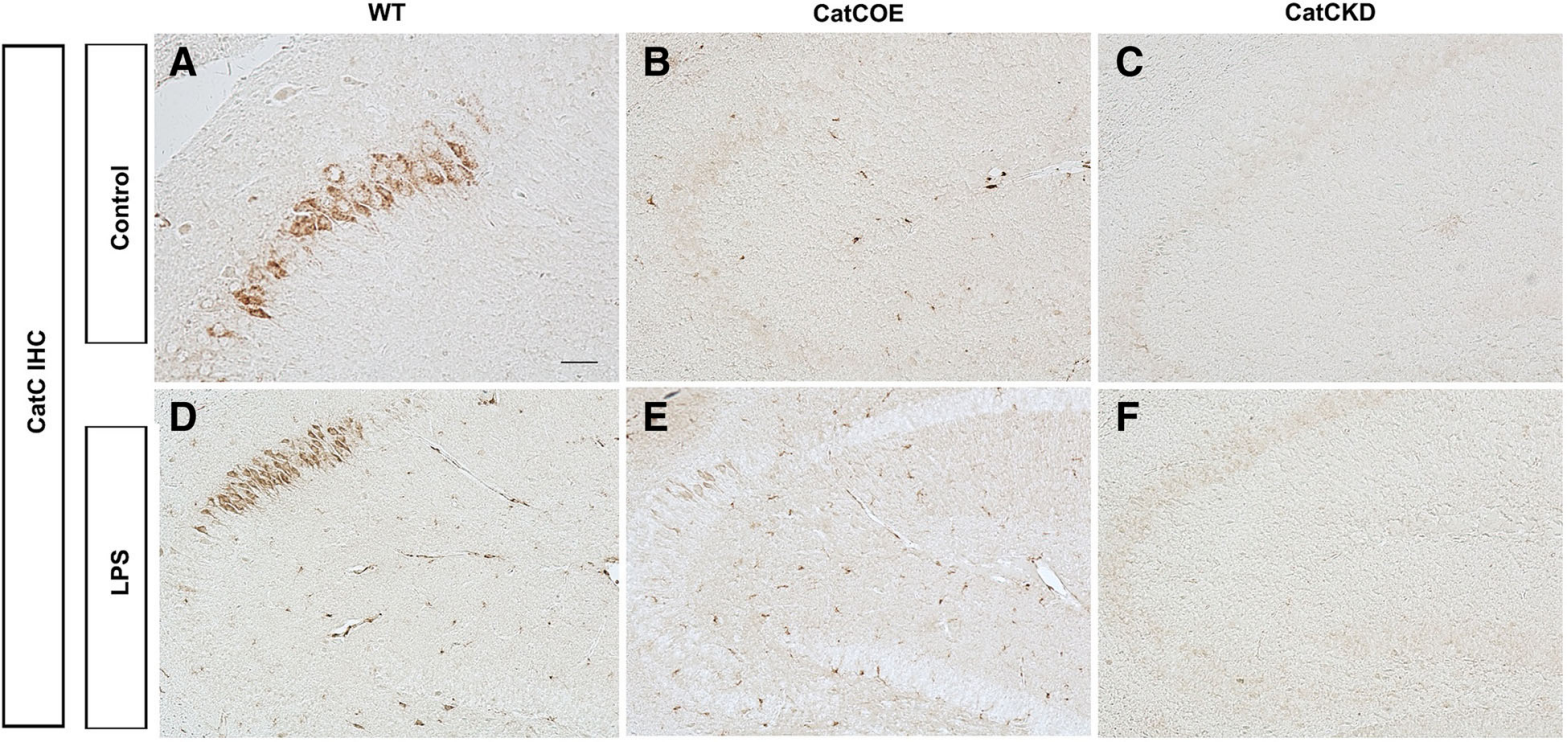
The expression of CatC in the transgenic mouse brain before and after administration of LPS. WT, CatCOE, and CatCKD mice were intraperitoneally injected with LPS (100 μg/kg) for 24 h before the brains were isolated. Immunohistochemical staining for CatC was performed on the frozen brain sections. LPS induced CatC expression in WT (**D**) and CatCOE (**E**) mice, but not in CatCKD (**F**) mice. Scale bars, 100 μm

In order to investigate the effects of CatC on neuroinflammatory status, we detected the expression of CatC in the wild-type, CatC OE, and CatC KD mice before and after administration of LPS (100 μg/kg, i.p.). The IHC staining results showed that CatC was predominantly expressed in hippocampal CA2 neurons in untreated WT mice, while in the other brain areas CatC expression was not detected (Fig. 2A). In CatC OE mice, CatC expression in hippocampal CA2 neurons disappeared, instead, it scattered almost throughout the brain (Fig. 2B). In contrast, no CatC-positive signals were found in CatC KD mouse brain (Fig. 2C). This pattern of CatC expression in transgenic mice confirmed the reliability of manipulation of CatC gene expression. Further, 24 h after LPS injection, CatC expression was increased in hippocampus and other brain areas in WT and CatC OE mice (Fig. 2D, E), but more obviously found in CatC OE mice. No CatC-positive signals were found in Cat KD mice after LPS injection (Fig. 2F).

**Fig. 3:**
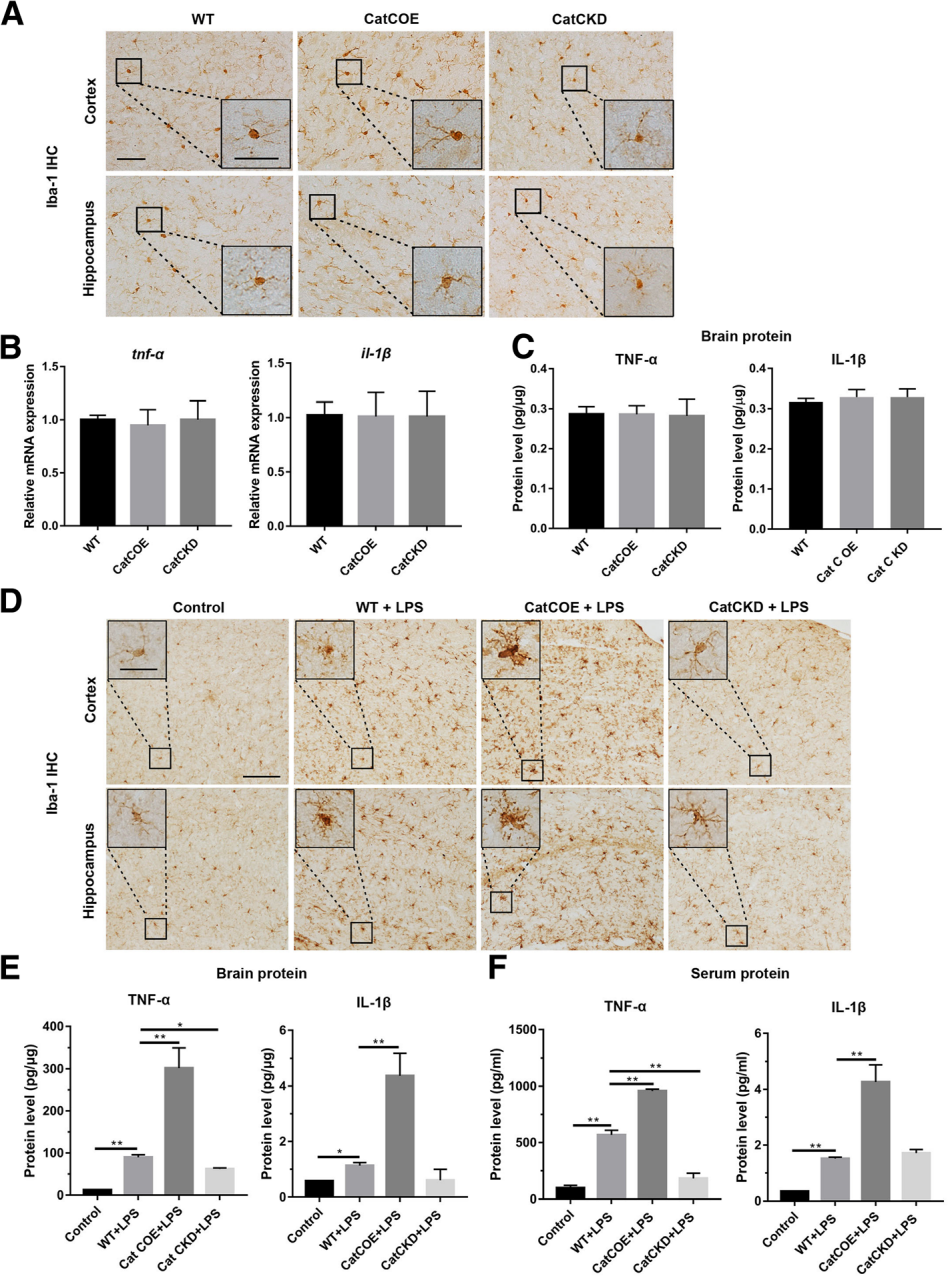
The effects of differential expression of CatC on microglia and proinflammatory cytokines in the brain. After spatial probe test, the activation of microglia was detected by Iba1 IHC staining in cortex and hippocampus of untreated mice (**A**) and LPS (i.p.)-injected mice (**D**). The levels of TNF-α and IL-1β were measured in untreated mice (**B**, **C**) and LPS-injected mice (**E**, **F**). The levels of TNF-α and IL-1β were determined by qRT-PCR and ELISA. Scale bars, 100 μm (main panels), 25 μm (insets). *P ≤ 0.05, **P ≤ 0.01. *n* = 3 (**A**–**D**), *n* = 3–5 (**E**, **F**)

In order to investigate neuroinflammatory status in CatC transgenic mice, MG activation and the expression of TNF-α and IL-1β were detected by Iba-1 immunohistochemical (IHC) staining, qRT-PCR, and ELISA. First, we assessed MG in untreated WT, CatCOE, and CatCKD mice. We found that the MG in the whole brain including cortex and hippocampus in CatCOE mice were slightly activated with slightly enlarged soma and shortened processes, compared with that in the WT and CatCKD mice (Fig. 3A). However, the expression of proinflammatory factors TNF-α and IL-1β did not exhibit significant differences among the WT, CatCOE, and CatCKD mice at mRNA (Fig. 3B) or protein level (Fig. 3C) in the whole brain, indicating that CatC differential expression did not affect the immune equilibrium in the brain in the condition of untreatment.

We next examined MG morphology and the expression of TNF-α, IL-1β in WT, CatCOE, and CatCKD mice treated with LPS (100 μg/kg, i.p.). After treatment for 24 h, mice were sacrificed, brains were removed, and serum was collected. The Iba-1 IHC results showed significantly activated MG with retracted processes, large cell bodies and nuclei in all the LPS-treated mice. Among them, more intensively activated patterns were found in CatCOE mice but weaker in CatCKD mice (Fig. 3D). Consistent with the activated status of MG, the expression of TNF-α and IL-1β in the brain (Fig. 3E) and serum (Fig. 3F) was significantly increased in the LPS-treated mice, and among them, the highest level was presented in CatCOE mice but lower levels in CatCKD mice. These results indicate that CatCOE aggravated LPS-induced inflammatory responses both in peripheral blood and the CNS.

### CatC aggravated neuroinflammation through accentuating MG towards M1 polarization following a central injection of LPS

**Fig. 4:**
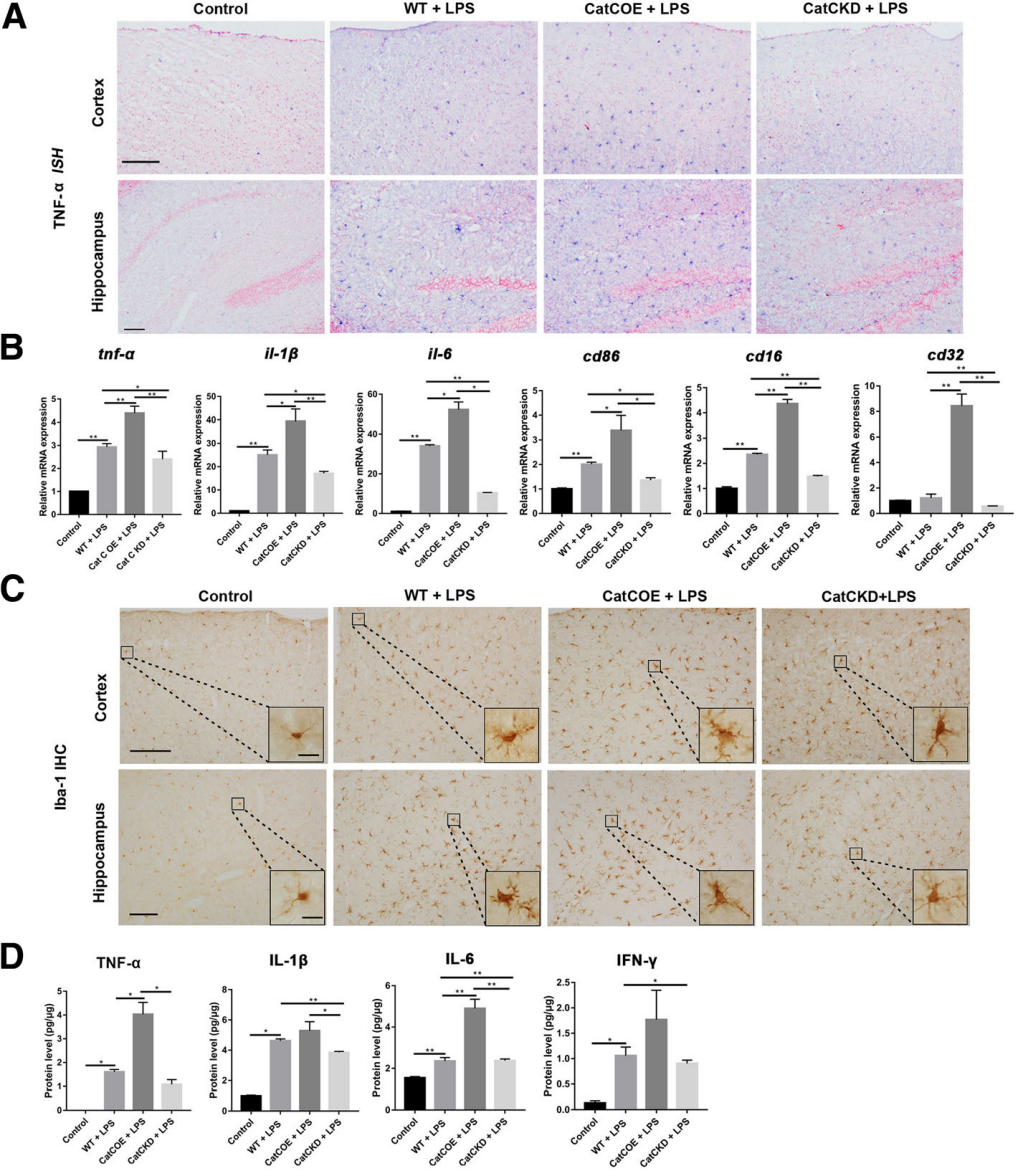
The effects of differential expression of CatC on M1 polarization of microglia in the brain. TNF-α *ISH* staining in cortex and hippocampus was performed 24 h after LPS (1 mg/kg, i.c.v.) injection (**A**). The levels of TNF-α and IL-1β were measured by qRT-PCR (**B**) and ELISA (**D**), respectively. The activation of microglia in cortex and hippocampus was detected by Iba1 IHC staining (**C**). Scale bars, 200 μm (main panels, first and second rows), 500 μm (main panels, third row), 100 μm (main panels, fourth row), 50 μm (insets). *P ≤ 0.05, **P ≤ 0.01. *n* = 3(**A**, **B**), *n* = 3–5 (**C**, **D**)

The mechanisms by which circulating LPS is transmitted into the CNS and causes neuroinflammation are not fully understood. Our previous findings and other studies suggested that LPS is able to activate the immune system by stimulation of monocytes, macrophages, neutrophils, blood platelets and endothelial cells [34, 35]. The activation of immune cells by LPS leads to release of inflammatory cytokines that are responsible for progression of inflammatory reactions and may cross the blood–brain barrier to mediate central effects. Thus, the increased proinflammatory cytokines in the brain could be derived from the activated MG, or from immune cells which are activated by systemic LPS administration in circulation, or both. In order to avoid the influence of systemic inflammation on the brain, we adopted LPS (1 mg/kg) i.c.v. injection to establish the neuroinflammation model. At 6 h post LPS i.c.v. injection, compared with that in untreated mice, the significantly increased expression of TNF-α and IL-1β mRNA was found in LPS-treated WT, CatCOE, and CatCKD mice by ISH staining (Fig. 4A) and qRT-PCR analysis (Fig. 4B). Among them, significantly higher expression was found in CatCOE mice compared with that in WT mice. In contrast, significantly alleviative expression was exhibited in CatCKD mice. Since the expression of TNF-α and IL-1β are closely associated with classical activation of MG (M1 phenotype), therefore, we evaluated mRNA expressions of other M1 markers including IL-6, CD86, CD16, and CD32 [36–38]. The mRNA expressions of these markers were very similar to those of TNF-α and IL-1β (Fig. 4B), suggesting that the inflammatory condition in CatCOE or CatCKD mice may be associated with MG activation status. We next evaluated the morphology of MG by Iba-1 IHC staining. As shown in Fig. 4C, although activated MG were found in the whole brain after LPS treatment, the most activated phenotypes of MG were found in CatCOE mice in both cortex and hippocampus. Also, the highest expression levels of TNF-α, IL-1β, and IL-6 protein were found in CatCOE mice, while the lowest level was found in CatCKD mice after LPS treatment (Fig. 4D). Taken together, these results suggested that CatC aggravated neuroinflammation induced by a central injection of LPS through accentuating MG towards M1 polarization.

### CatC increased production of proinflammatory factors under LPS stimulation by accentuating MG towards M1 polarization

**Fig. 5:**
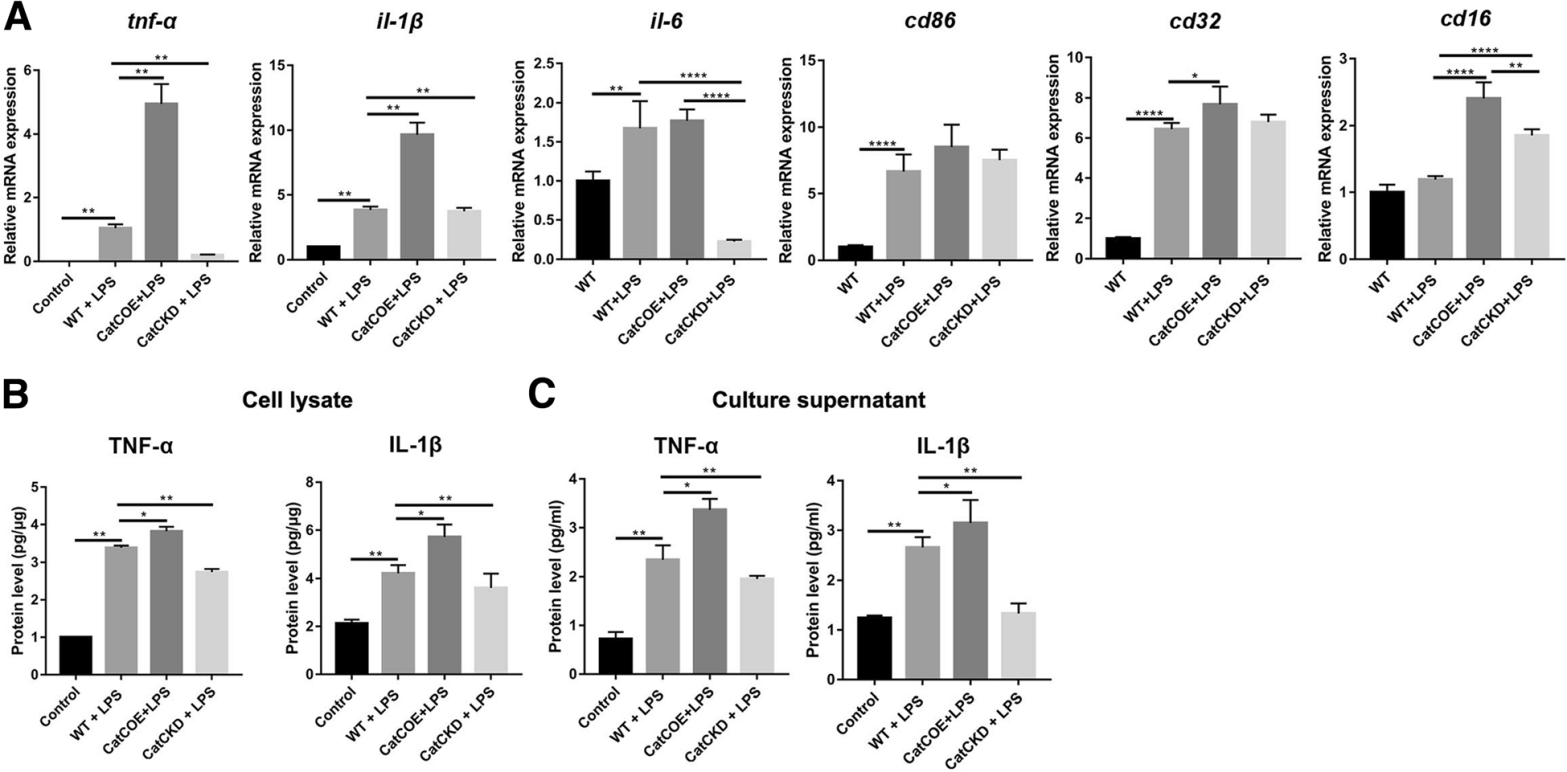
The effects of differential expression of CatC on M1 polarization of microglia in vitro. Primary cultured microglia from WT, CatCOE, and CatCKD mice were stimulated with LPS (50 ng/ml) for 24 h. **A** The mRNA expressions of TNF-α, IL-1β, IL-6, CD86, CD32, and CD16 were measured by qRT-PCR. **B** The levels of TNF-α and IL-1β in cellular lysate and culture supernatant were measured by ELISA. *P ≤ 0.05, **P ≤ 0.01, ****P ≤ 0.0001

In order to confirm our speculation, we performed primary MG culture from WT, CatCOE, and CatCKD mice and stimulated MG with LPS (50 ng/ml) for 24 h. We first detected mRNA expression of M1 markers including TNF-α, IL-1β, IL-6, CD86, CD32, and CD16. The results were very similar to those in in vivo, with the highest levels exhibited in MG of CatCOE mice, while the lowest in MG of CatCKD mice (Fig. 5A), suggesting CatC accentuated MG towards M1 polarization after LPS treatment. Then ELISA was carried out to detect the expression of TNF-α and IL-1β protein in cell lysates (Fig. 5B) and culture supernatants (Fig. 5C). The results showed that the highest levels of IL-1β and TNF-α were found in CatCOE MG in both cell lysates and culture supernatants. In contrast, CatCKD MG exhibited the lowest levels. These results further suggested that CatC aggravated LPS-induced inflammation through accentuating MG to M1 polarization in vivo.

### CatC stimulation promoted MG towards M1 phenotype in vitro and in vivo

**Fig. 6:**
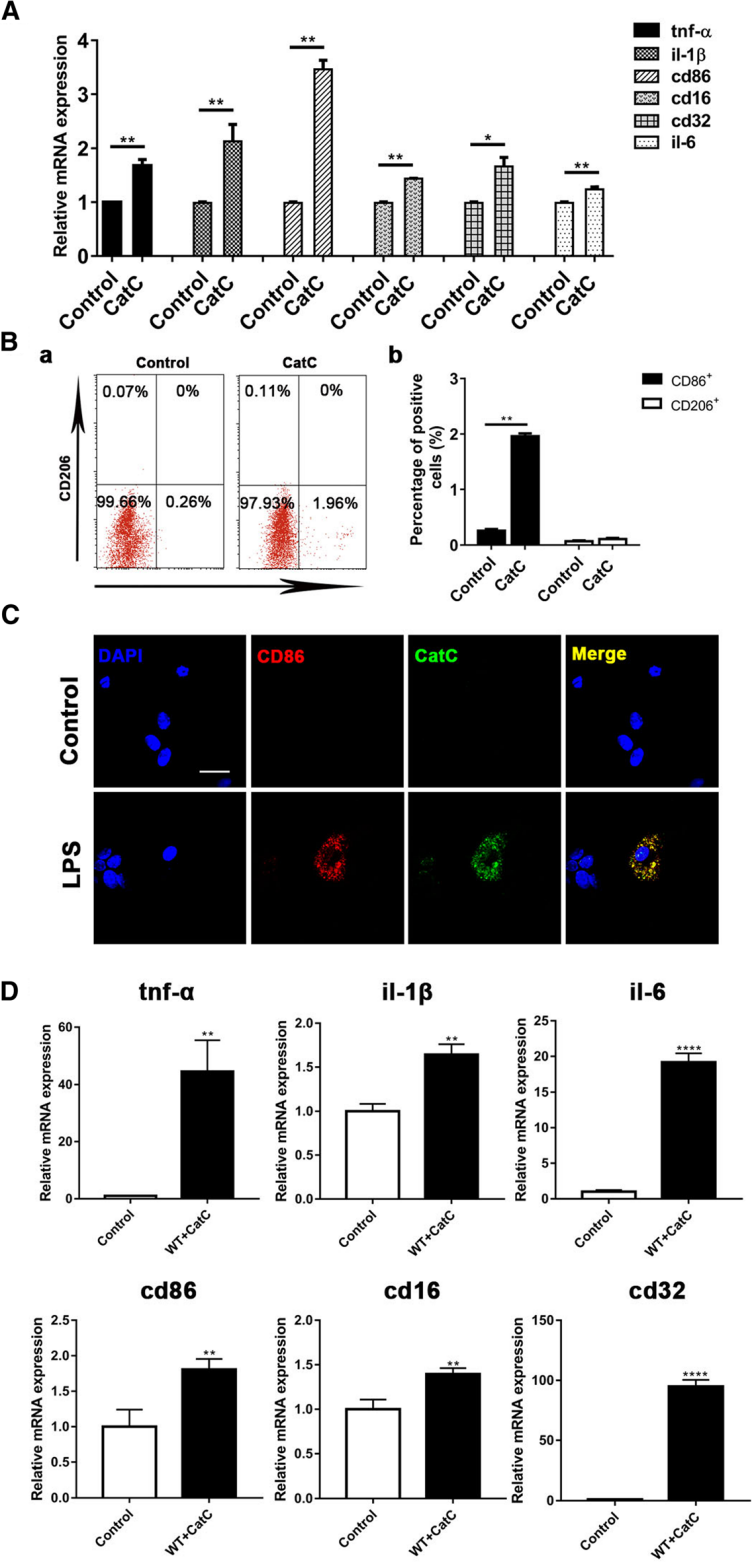
The effects of CatC stimulation on the expression of M1 markers of microglia in vitro. Primary cultured microglia from WT mice was stimulated with active CatC (10 ng/ml) for 18 h. **A** The mRNA expressions of TNF-α, IL-1β, CD86, CD16, CD32, and IL-6 were measured by qRT-PCR. **B** The percentages of CD86^+^ and CD206^+^ cells were measured by flow cytometry. **C** CD86 and CatC co-expression was detected by immunofluorescent staining after LPS (50 ng/ml) treatment for 18 h. **D** The mRNA expressions of TNF-α, IL-1β, CD86, CD16, CD32, and IL-6 were measured by qRT-PCR in mice with injection of CatC (0.43 mg/μl, i.c.v.) for 6 h. Scale bars, 25 μm. The data represent mean ± SEM, *n* = 3. *P < 0.05, ** *P* < 0.01, ****P < 0.0001

It has been well known that LPS can activate MG and promote them to polarize to M1 activation status, producing proinflammatory factors and aggravating neuroinflammation [39–41]. In our present study, both in vivo and in vitro results suggested that CatC enhanced MG activation and production of IL-1β and TNF-α under LPS stimulation. And more importantly, the mRNA expressions of M1 markers were upregulated in CatCOE mice and cultured CatCOE MG while downregulated in CatCKD mice and cultured CatCKD MG, suggesting that CatC accentuated MG towards M1 polarization under LPS stimulation. In our previous study, we reported that CatC is expressed intracellularly and secreted extracellularly in MG following LPS stimulation [17]. Therefore, we speculate whether exogenous CatC can directly facilitate MG towards M1 activation status. We used active CatC (10 ng/ml) to stimulate primary cultured MG from WT mice. After 18 h stimulation, we found that the mRNA expression of M1 markers for MG including TNF-α, IL-1β, CD86, CD16, CD32, and IL-6 were significantly elevated compared with that in untreated MG by qPCR (Fig. 6A). We further used CD86 and CD206 as the markers of M1 and M2 phenotypes to perform flow cytometry analysis after exogenous CatC stimulation of MG. The results showed that the number of CD86 positive cells was increased significantly in CatC-treated MG (Fig. 6B (a, b)). These results suggested that exogenous CatC alone, independent of LPS, could promote MG polarized towards M1 phenotype. In our previous study, we reported that LPS could induce CatC expression in cultured MG, and it is known that LPS is one of the inducers for M1 polarization, so we wondered whether CatC-expressing MG was M1 phenotype. Therefore, we performed CD86 and CatC double immunofluorescence staining in LPS-treated MG, and untreated MG was used as the control (Fig. 6C). As we expected, both CD86 and CatC-positive MG were only found in LPS-treated MG, suggesting CatC could not only promote MG towards M1 phenotype but also be produced by MG of M1 phenotype.

In order to confirm these results in vivo, we performed CatC (10 ng/ml) i.c.v. injection, and equivolume 0.9% saline vehicle injection mice were used as the control. After 6-h treatment, total mRNA was isolated from the whole brain, and mRNA expressions of aforementioned M1 markers were detected by qRT-PCR (Fig. 6D). The results showed that mRNA expression of all the markers were significantly higher than that in control mice. Taken together, in vivo and in vitro the results suggest that exogenous CatC facilitate MG towards M1 activation status.

### Exogenous CatC caused numerous gene expression changes including NR2B which is the subunit of NMDA receptors

**Fig. 7:**
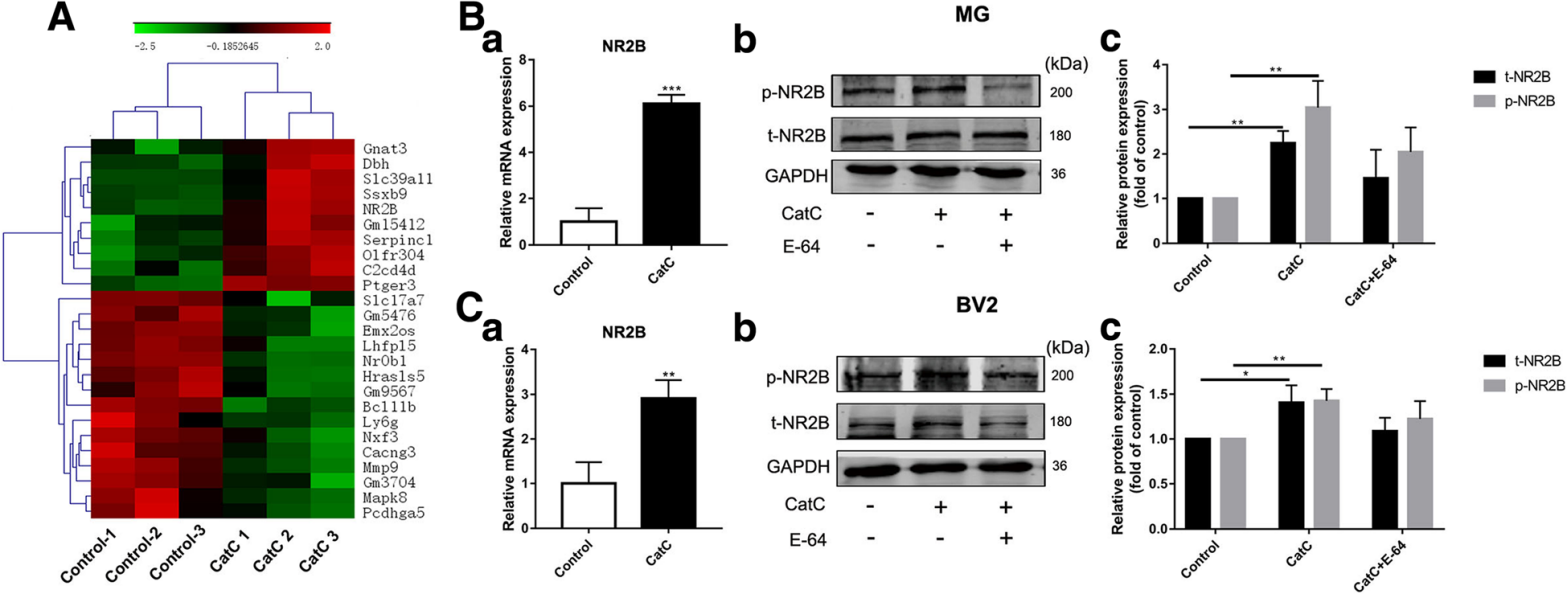
The changes of expression of NR2B in CatC-stimulated microglia in vitro. Primary cultured microglia form WT mice were stimulated with 100 ng/ml active CatC for 24 h, then microarray-based gene expression analysis was performed. **A** The heat map showed 25 differentially expressed genes, including 10 upregulated genes and 15 downregulated genes. **B** The mRNA expression of NR2B was verified by qRT-PCR with 100 ng/ml active CatC stimulation and/or cathepsin inhibitor E-64 (50μΜ) for 12 h (a). Phosphorylated NR2B (p-NR2B) and total NR2B (t-NR2B) expressions were quantified in CatC-stimulated microglia by western blot analysis (b, c). **C** The same experiments were performed in BV2 cells. GAPDH as a loading control. The data represent the increased folds of treatment groups relative to the control. The data represent mean ± SEM, *n* = 3. *P < 0.05, **P < 0.01, ***P < 0.001

So far, we have demonstrated that CatC can aggravate neuroinflammation through inducing MG towards M1 phenotype. To identify the underlying molecular changes of CatC promoting MG towards M1 phenotype, microarray-based gene expression analysis was performed on primary cultured WT MG stimulated with active CatC (100 ng/ml, 12 h) for 24 h. After that, total RNA was extracted, and the genomic expression profile of CatC after MG treatment was analyzed. Microarray hybridization signal scanning showed that fluorescence signaling quality control was good, and the ratio of signal to noise was low (Data not shown). Hierarchical cluster analysis of differentially expressed (DE) genes showed that the genes ultimately clustered into two major branches, indicating significant differences between untreated and CatC-treated MG. Results showed that there were 254 DE genes in CatC-stimulated MG, including 103 upregulated genes and 151 downregulated genes. As shown in Fig. 7A, the heat map includes 10 upregulated genes and 15 downregulated genes. Upregulated genes are shown in red and downregulated genes are in green. Combining the results of GO (Gene Ontology) analysis, NR2B was chosen as a molecule for in-depth study.

Therefore, we detected expression of NR2B in both primary cultured MG and BV2 cells with CatC stimulation. qRT-PCR results showed that the mRNA expression of NR2B was increased in CatC-treated primary cultured MG (Fig. 7B (a)) and BV2 cells (Fig. 7C (a)). The expression of phosphorylated NR2B (p-NR2B) and total NR2B (t-NR2B) was also increased in CatC-treated primary cultured MG (Fig. 7B (b, c)) and BV2 cells (Fig. 7C (b, c)). Moreover, we inhibited CatC activity by using cathepsins inhibitor E-64. Western blot analysis showed that there was lowered tendency for expression of p-NR2B and t-NR2B in E-64 and CatC co-incubated primary cultured MG (Fig. 7B (b, c)) and BV2 cells (Fig. 7C (b, c)) compared with that in CatC-treated alone, which may be associated with inhibitory effects of E-64 on CatC activity. These data suggest that exogenous CatC stimulation increased the expression of activated NR2B.

### CatC induced activation of Ca^2+^-dependent PKC/p38 MAPK/NF-κB signaling pathway in MG

NR2B is the regulation subunit of N-methyl-D-aspartate receptors (NMDARs) which are ligand-gated ion channels with high permeability to Ca^2+^ [42], a crucial intracellular second messenger. Intracellular Ca^2+^ overload may activate a variety of intracellular signal transduction pathways [43], including Ca^2+^-dependent PKC/p38 MAPK/NF-κB cascades to induce MG activation, control a range of cellular process, including chemotaxis, phagocytosis, and secretion of cytokines.

**Fig. 8:**
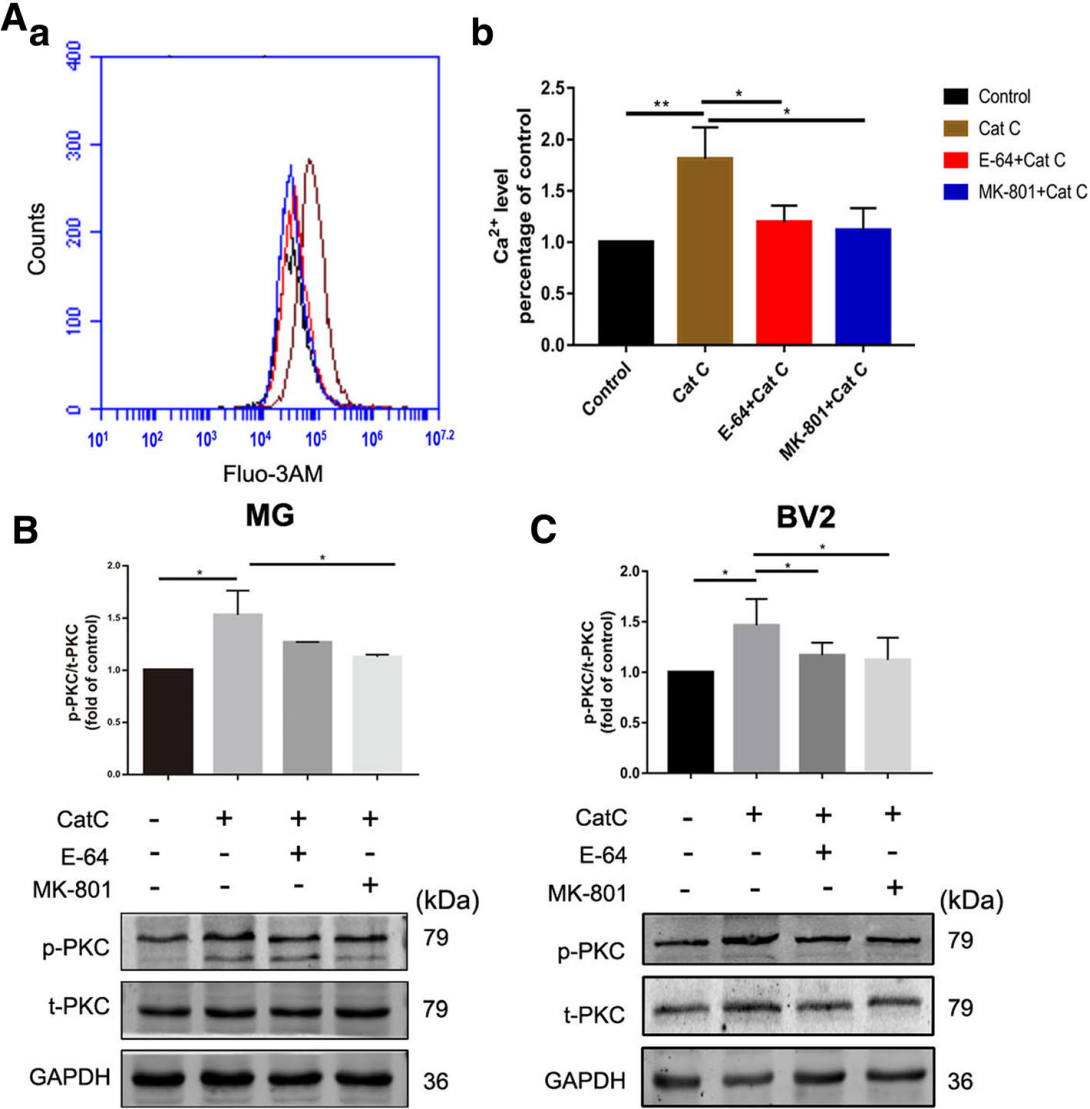
CatC stimulation increased the concentration of intracellular Ca^2+^ and induced PKC activation. BV2 cells were stimulated with active CatC (100 ng/ml) and co-stimulated with E-64 (50 μM) or pre-incubated with MK-801 (10 μM) for 6 h. Then, intracellular Ca^2+^ concentration was measured by flow cytometry (**A**). Primary cultured microglia form WT mice and BV2 cells were stimulated with active CatC (100 ng/ml), or co-stimulated with E-64 (50 μM), or treated with MK-801 (10 μM) prior to CatC stimulation for 12 h. The expressions of p-PKC and t-PKC were quantified by western blot analysis in primary cultured microglia (**B**) and BV2 cells (**C**). The bands shown are representative of three independent experiments. GAPDH as a loading control. The statistics are shown as increased folds of treatment groups to the control. The data represent mean ± SEM, *n* = 3. *P < 0.05, **P < 0.01

**Fig. 9:**
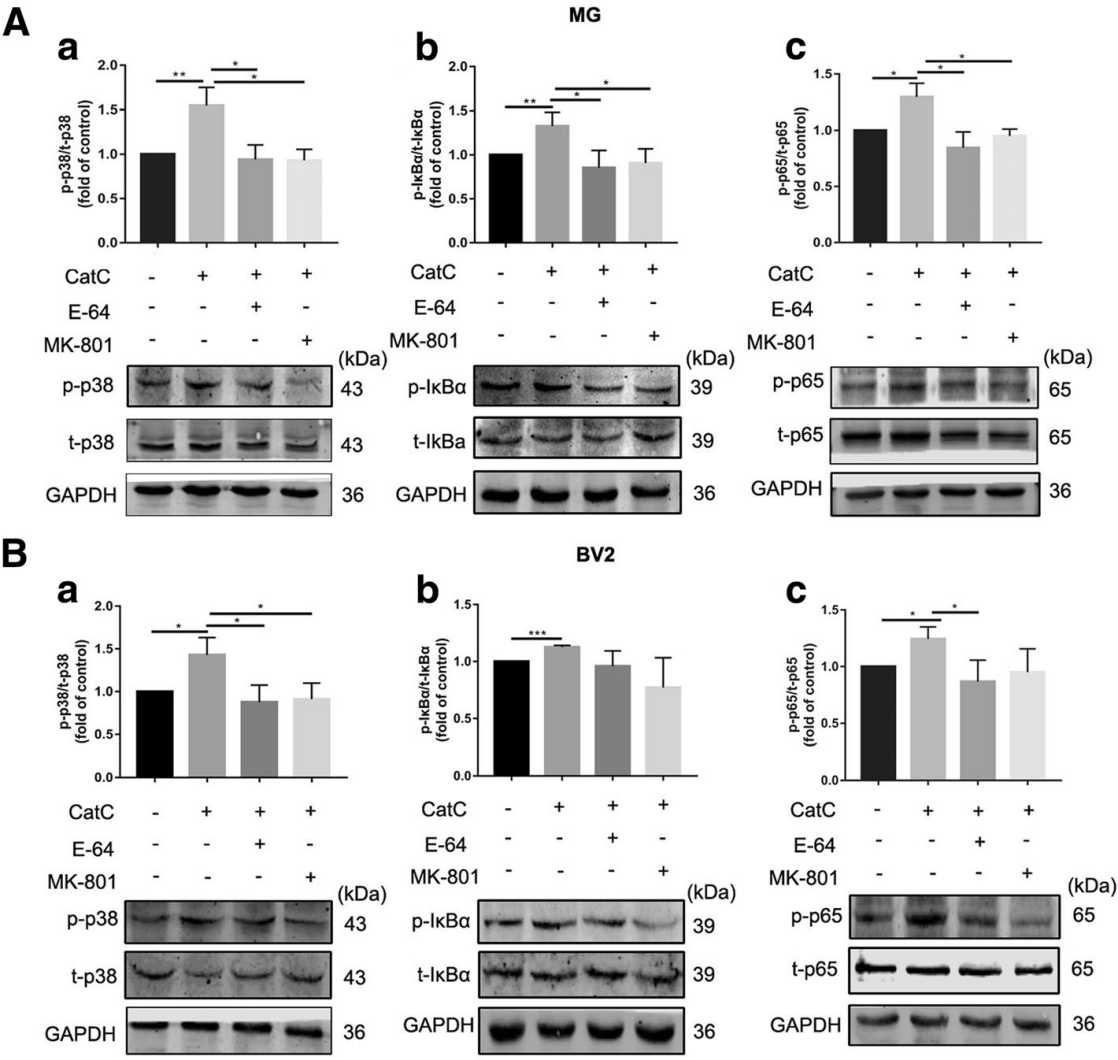
CatC activated p38 MAPK/NF-κB signaling pathway in microglia. **A** Primary cultured microglia from WT mice were treated with active CatC (100 ng/ml), or co-stimulated with E-64 (50 μM), or pretreated with MK-801 (10 μM). The expressions of p-p38, t-p38, p-IκBα, t-IκBα, p-p65, and t-p65 were quantified by western blot analysis. The levels of p-p38/t-p38 (a), p-IκBα/t-IκBα (b), p-p65/t-p65 (c) were shown as increased folds of treatment groups relative to control group. **B** Activation of p38 MAPK/NF-κB signaling pathway was examined in BV2 cells. The bands shown are representative of three independent experiments. The data represent mean ± SEM, *n* = 3. *P < 0.05, **P < 0.01

In the present study, we have found that CatC promoted MG towards M1 phenotype and the expression of NR2B. To confirm that the effects of CatC on MG is mediated by NMDAR-mediated Ca^2+^ signals, the intracellular Ca^2+^ concentration was measured by flow cytometry. As shown in Fig. 8A, fluorescence of the Ca^2+^ indicator Fluo-3 AM in CatC-treated BV2 cells was increased compared with the control, and this response was reversed by addition of E-64 and NMDAR antagonist MK-801, respectively (Fig. 8A). The data indicate that the elevated Ca^2+^ levels in the cytoplasm following CatC treatment was mediated by NMDAR. Furthermore, western blot analysis showed that CatC stimulation (100 ng/ml, 12 h) induced a significant increase of PKC phosphorylation compared with the control in both primary cultured MG (Fig. 8B) and BV2 cells (Fig. 8C). In addition, the phosphorylation of p38 (Fig. 9A (a), B (a)), IκBα (Fig. 9A (b), B (b)) and p65 (Fig. 9A (c), B (c)), the key molecules of MAPKs/NF-κB pathway, were increased in CatC-treated primary cultured MG (Fig. 9A) and BV2 cells (Fig. 9B), while addition of E-64 and MK-801 reversed the phosphorylation of above molecules. Together, the data suggest that CatC enhanced NR2B expression and further induced the activation of Ca^2+^-dependent PKC/p38 MAPK/NF-κB pathway, leading to an increase in production and release of proinflammatory cytokines in MG.

## Discussion

In the present study, differential expression of CatC produced different effects on MG activation, cytokine expression and spatial learning, and memory in CatCOE, CatCKD, and wild-type mice pre-treated with LPS centrally or peripherally. CatC aggravated neuroinflammation by promoting MG polarization towards M1 phenotype. This effect is mainly achieved through activation of Ca^2+^-dependent PKC/p38 MAPK/NF-κB pathway which is probably triggered by glutamate receptor subunit NR2B. To the best of our knowledge, this is the first study to demonstrate the possible molecular mechanisms of CatC-involved neuroinflammation in the brain.

Chronic neuroinflammation is ubiquitous pathological change in many neurological diseases, such as neurodegenerative diseases, and mood-related diseases. In these diseases, inflammation responses present in a prolonged and persistent pattern, leading to progressive changes of the inflammatory process in neural tissues. The mechanisms underlying maintenance of chronic neuroinflammation remains unclear. It has been found that chronically activated MG adversely secrete mediators to promote inflammation and damage neurons. Microglia-driven neuroinflammation has been confirmed to contribute to the progression of neurological diseases [44–46]. MG express and secrete several different cathepsins, a large group of lysosomal proteases, to participate in the key neuroinflammatory pathways and processes [47–53], supporting various immune functions of MG [54]. CatC is synthesized as inactive preprocathepsin C in vivo and is processed into its active mature form by a series of proteolytic cleavages. Some endopeptidases, presumably cathepsin L or S, are responsible for the activation of CatC [55]. Once activated, CatC mediates the conversion of granule serine proteases from their inactive (zymogen) into the enzymatically active protease by removing an N-terminal propeptide [10].

In the present study, CatC overexpression enhanced MG towards M1 polarization, induced proinflammatory cytokines expression in LPS-pretreated mice, and in vitro CatC alone induced the same effects on MG polarization. Our previous study found that the CatC expression was increased in a dose-dependent manner in lysates and medium of cultured MG treated with LPS, IL-1β, or IL-6. Meanwhile, enzymatic activity of CatC was also upregulated. These findings indicate that inflammatory stimuli can induce the expression and release of CatC in MG. Herias et al. [20] reported that CatC may exert immunomodulatory effects on macrophages through an autocrine positive feedback of CatC in macrophage polarization towards M1 in atherosclerotic lesion. Collectively, this evidence strongly points to a potential interaction between inflammatory cytokines and CatC in MG, and such interactions contribute to amplify cytokines and neuroinflammation-involved cellular events, responsible for the continuous inflammatory cycle accompanying pathogenesis in the CNS.

It should be noted that in the absence of exogenous stimuli, differential expression of CatC did not affect learning and memory ability or the expression of cytokines, except that slight activation of MG was observed in CatCOE mice. Overall, immune homeostasis was maintained in vivo, regardless of CatC expression. However, upon immune activation, just like LPS injection in the present study, CatC will enter inflammatory cycle to further aggregate the inflammatory responses.

NMDAR is a heteromeric ligand-gated ion channel that interacts with multiple intracellular proteins by way of different subunits [56]. The functional NMDAR expression is detected in membrane fraction of MG, and stimulation of NMDAR can trigger activation of MG and secretion of inflammatory factors in vitro [57]. Also, brain inflammation affects the expression of NMDAR in the hippocampal areas [58]. These findings point to the potential roles of NMDAR in MG-mediated inflammation. In our study, microarray-based gene expression analysis revealed that CatC stimulation caused an increase in mRNA expression of NMDAR subunit, NR2B. Therefore, NR2B was selected to be a downstream effector gene following CatC treatment. NR2B has been demonstrated to play unique/critical roles in determining the structural and functional properties of the NMDAR. The changes in brain environment resulting from kinases, phosphatases, and other regulatory enzymes can influence the expression, distribution, and function of the NR2B subunit. The inflammatory stimuli had been shown to promote NR2B expression in the brain. Maher et al. [59] reported that NR2B is overexpressed in systemic LPS-induced AD model mice, and increased NO concentration in the brain may contribute to NR2B expressional upregulation. Harré et al. [60] showed a pronounced long-term upregulation of NR2B mRNA in the hippocampus and cortex of rats following systemic LPS administration. Consistently, our study also showed that LPS-treated MG produced a higher level of NR2B mRNA compared to untreated cells (data not shown). LPS is a well-documented strong inducer for MG M1 polarization, and CatC has been proven to have the same effect on MG. The fact that LPS and CatC both enhanced NR2B transcripts in MG drives us to suppose that the upregulation of NR2B transcript following CatC treatment may be associated with increased proinflammatory cytokine release derived from M1 phenotype of MG. Our data not only indicate that NR2B is related to immune activation of MG, but also establish the potential functional links between CatC and NR2B in MG. However, how and whether CatC modulates directly the functional expression of NR2B and further affects receptor function in MG is yet to be understood.

NR2B subunit is composed of 1456 amino acids with an approximate molecular mass of 170–180 kDa, possessing extracellular NH2-terminal signal peptide, intracellular C-terminal domains and 4 putative transmembrane domains (M1–M4) [42, 61]. In the second transmembrane region, an asparagine residue is the putative pore-forming region and play a role in the high Ca^2+^ permeability of the channel. Previously, Murugan et al. [62] reported that activated MG expressed functional NMDAR. A notable increase in intracellular calcium was observed after NMDAR activation in hypoxia, which was significantly reduced by addition of MK801 (a NMDA channel blocker) in primary cultured MG. Moreover, the hypoxia-induced activation of NF-κB signaling pathway in MG was suppressed by administration of MK801. In our study, in primary cultured MG, an increase in intercellular Ca^2+^ levels was blocked by MK801, indicating potentiation of Ca^2+^ influx following CatC treatment was mediated by NMDAR, most probably by increased NR2B. The intracellular Ca^2+^ overload may induce PKC activation, leading to inflammatory response by activation of p38 MAPK-dependent NF-ĸB pathway [44].

## Conclusions

In conclusion, CatC induced NR2B expression in MG, further activated Ca^2+^-dependent PKC/p38 MAPK/NF-κB pathway, and promoted M1 polarization of MG, leading to aggravation of neuroinflammation. The interactions between CatC and inflammatory cytokines in MG may be one of the main reasons of persistent neuroinflammation in CNS. Our data suggest that CatC may be one of key molecular targets for alleviating and controlling neuroinflammation in the neurological diseases.

## Abbreviations

BCIP: 5-bromo-4-chloro-3-indolyl-phosphate
CatC: Cathepsin C
CNS: Central nervous system
COPD: Chronic obstructive pulmonary disease
CXCL2: Chemokine (C-X-C motif) ligand 2
DE: Differentially expressed
DMEM: Dulbecco’s modified Eagle’s medium
ELISA: Enzyme-linked immunosorbent assay
FBS: Fetal bovine serum
i.c.v.: Intracerebroventricular
i.p.: Intraperitoneal
IHC: Immunohistochemical
IL-1β: Interleukin-1β
IL-6: Interleukin-6
KD: Knock down
LPS: Lipopolysaccharides
LTP: Long-term potentiation
MG: Microglia
MWM: Morris water maze
NBT: 4-nitro blue tetrazolium chloride
NMDAR: N-methyl-D-aspartate receptor
OE: Over expression
qRT-PCR: Quantitative real-time polymerase chain reaction
TNF-α: Tumor necrosis factor-α
