# Correction to: Cathepsin C promotes microglia M1 polarization and aggravates neuroinflammation via activation of Ca^2+^-dependent PKC/p38MAPK/NF-κB pathway

**DOI:** 10.1186/s12974-021-02346-1

**Published:** 2022-01-11

**Authors:** Qing Liu, Yanli Zhang, Shuang Liu, Yanna Liu, Xiaohan Yang, Gang Liu, Takahiro Shimizu, Kazuhiro Ikenaka, Kai Fan, Jianmei Ma

**Affiliations:** 1 Department of Anatomy, Dalian Medical University, West Section No.9, South Road, Lvshun, Liaoning, 116044 Dalian China; 2 Liaoning Provincial Key Laboratory of Brain Diseases, Dalian Medical University, Liaoning, 116044 Dalian China; 3 Basic Medicine College, Dalian Medical University, Liaoning, 116044 Dalian China; 4 Wolfson Institute for Biomedical Research, University College London, London, UK; 5 Division of Neurobiology and Bioinformatics, National Institute for Physiological Sciences, Okazaki, Aichi 444-8787 Japan; 6 The National and Local Joint Engineering Research Center for Drug Development of Neurodegenerative Disease, Dalian Medical University, Dalian 116044 Liaoning, China

## Correction to: Journal of Neuroinflammation (2019) 16:10 https://doi.org/10.1186/s12974-019-1398-3

The original version of this article [1] unfortunately contained a mistake.

In recent research work, the mistake occurred on primer sequence of NR2B for RT-PCR shown in Table 1 (Material and method).

The corrected primer sequence of NR2B should be as followings

Forward: GCTCATCGCCAAGGGTACATC

Reverse: TGCACTATTTCAAGTCACATGCCTA

The correct version of Table 1 is given here.

**Table 1:** Primer sequences used for RT-PCR analysis

Gene	Primer	
	Forward	Reverse
TNF-α	ATCCGCGACGTGGAACTG	ACCGCCTGGAGTTCTGGAA
IL-1β	GAGCACCTTCTTTTCCTTCATCTT	TCACACACCAGCAGGTTATCATC
IL-6	CAACGATGATGCACTTGCAGA	CTCCAGGTAGCTATGGTACTCCAGA
iNOS	TAGGCAGAGATTGGAGGCCTTG	GGGTTGTTGCTGAACTTCCAGTC
CD16	GCCAATGGCTACTTCCACCAC	GTCCAGTTTCACCACAGCCTTC
CD32	CCAGAAAGGCCAGGATCTAGTG	GGGAACCAATCTCGTAGTGTCTGT
NR2B	GCTCATCGCCAAGGGTACATC	TGCACTATTTCAAGTCACATGCCTA
Ptk2	AGAACTTGGACGCTGTATTGGAGA	TGCAACAGCCAAAGCTGGA
Ptger3	TGTGTGCTGTCCGTCTGTTG	CTTCTCCTTTCCCATCTGTGTCTT
Slamf8	GTGCCAATTACACTGTTCCTGATCC	GTCCAAGCACCAGTTTATGTTGTCC
Pex5l	CCAACACTTTCATATCCGTTGCTC	CTGCCTCTGGCATGGTTTCA
HPSE	ACAAACGGAGTTGTTGAAGTGAGG	AGCACTACAGACATCGGGACAGAG
β-actin	TCATCACTATTGGCAACGACG	AACAGTCCGCCTAGAAGCAC
